# Preparation of Herbal Tea as Infusion or by Maceration at Room Temperature Using Mistletoe Tea as an Example

**DOI:** 10.3797/scipharm.1006-06

**Published:** 2010-11-20

**Authors:** Sebastian Jäger, Markus Beffert, Katharina Hoppe, Dominik Nadberezny, Bruno Frank, Armin Scheffler

**Affiliations:** 1 Carl Gustav Carus-Institute, Am Eichhof 30, 75223, Niefern-Öschelbronn, Germany; 2 Betulin-Institute, Blumenstr. 25, 64297, Darmstadt, Germany; 3 Kneipp-Werke, Steinbachtal 43, 97082, Würzburg, Germany

**Keywords:** Herbal tea, Mistletoe, *Viscum album* L, Infusion, Maceration, Extraction Chromatographic profile, HPLC, GC, ELISA

## Abstract

Herbal tea can be prepared by infusion or maceration at room temperature resulting in different compositions of extractable constituents, which possibly influences the mode of action or safety profile. Knowledge on this topic is limited. The aim of this study was to investigate the substantial differences between infusion and maceration as recommended preparation methods for the preparation of herbal mistletoe tea, a traditional remedy against cardiovascular diseases. No active substances are known but analytical marker substances such as proteins, triterpenoids, phenylpropane derivatives and flavonoids can be quantified within the herb and the different herbal tea preparations. Whereas phenylpropane derivatives were completely extracted by infusion and maceration, neither method dissolved viscotoxins. 43% of mistletoe lectins were extracted by maceration, whereas by infusion they are inactivated by thermal degradation. By contrast, oleanolic acid and betulinic acid are present in higher concentrations in infusates compared with macerates, but even infusion extracted less than 2%. Infusion extracted 43% of flavonoid-like substances and maceration only 31%. In conclusion this study determines some differences between both extraction methods on the profile of solved substances. The relevance of it should be determined in studies dealing with the efficacy of herbal mistletoe tea.

## Introduction

Herbal tea is mostly prepared by infusion, but in rare cases maceration at room temperature (r.t.) is required. *Althaea officinalis* L. is an example. Its starch agglutinates with heating so maceration at r.t. is used for the preparation of its herbal tea [1]. Knowledge on this topic is limited. This is why the European committee on herbal medicinal products within the European Medicines Agency states (04.09.2008, Doc. Ref. EMEA/HMPC/451978/2008) that ‘the method of preparation and the concentration of the herbal tea may be crucial, because different preparations may exert different actions and in some circumstances may have a different safety profile’. An example of where both preparation methods are described is traditional herbal mistletoe tea for cardiovascular diseases [2]. Nowadays the manufacturers of herbal mistletoe tea recommend the preparation by infusion but Wichtl published 2009 the preparation by maceration with cold water for 12 hours [3], but the compositional difference between the resulting preparations is as yet unknown. The use of mistletoe *Viscum album* L. as a remedy has an ancient tradition in treating various diseases [4]. Using different methods of manufacturing the resulting dosage form ranges from orally applied herbal teas, to dragées, to drops and to extracts suitable for injection [5]. Based on tradtiton mistletoe teas are used in the treatment of hypertension, cardiovascular diseases and arthritis. Nevertheless mistletoe constituents were reviewed for their pharmacological properties [6]. Viscotoxins (VT) and flavonoids may play a role at hypertension [6]. Syringin (SY, eleutheroside B) and syringenin apiosylglucoside (SYA) are suitable analytical marker substances, but their cardiovascular properties are controversial [7–11]. The use of mistletoe for arthritis has not yet been correlated with its component substances, but anti-cancer properties have been partly correlated with certain proteins and triterpenoids [6]. VT and mistletoe lectins (ML) act as necrotic and apoptotic proteins, whereas the terpenes oleanolic acid (OA) and betulinic acid (BA) posses apoptotic as well as anti-angiogenic, anti-inflammatory and redifferentiating properties [6, 12]. Because of their structural diversity, the extraction of mistletoe constituents is dependent on the extraction process. For instance, the low aqueous solubility of OA and BA is well known, so too the poor solubility of ML at acidic pH values [5, 13]. Herbal mistletoe tea is recommended to be prepared as an infusate or by maceration at r.t. [2, 3] But the influence of these two techniques on the profile of extracted substances is unknown. A first step to improve the evidence of herbal teas could be the determination of the solved substances. Mistletoe tea is a suitable example because analytical methods for structural different substances are known. This is why this study was undertaken with mistletoe tea to determine the influence of the extraction process on the extraction of the marker substances VT, ML, OA, BA, β-amyrin acetate (bAA), lupeol acetate (LA), SY, SYA and flavonoid like substances (flavonoid fingerprint at 360 nm).

## Results and Discussion

**Fig. 1.**
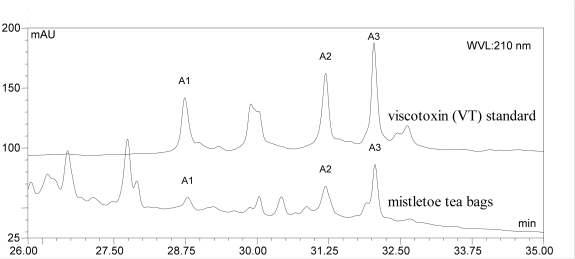
HPLC chromatogram (210 nm) of VT isoforms A1, A2 and A3 in plant material (mistletoe tea bags).

**Fig. 2.**
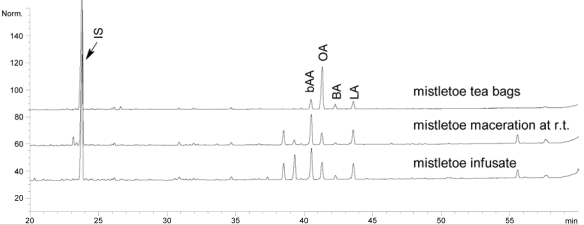
GC chromatogram of triterpenoids in plant material (mistletoe tea bags), herbal mistletoe maceration at r.t. and within infusate. IS…internal standard cholesterol; bAA…β-amyrin acetate; OA…oleanolic acid; BA…betulinic acid; LA…lupeol acetate.

**Fig. 3.**
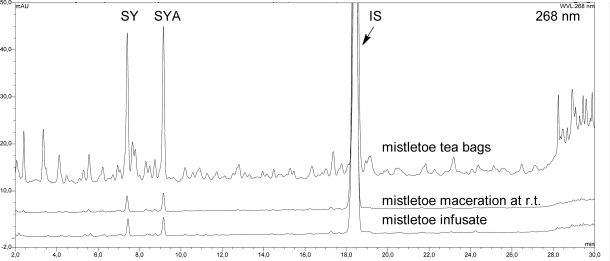
HPLC chromatogram (268 nm) of syringin (SY) and syringenin apiosylglucoside (SYA) in plant material (mistletoe tea bags), herbal mistletoe maceration at r.t. and within infusate. Phenacetin was used as internal standard (IS).

**Fig. 4.**
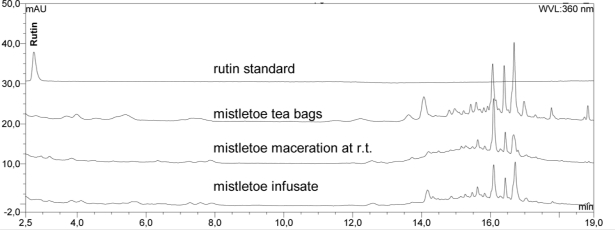
HPLC fingerprint (360 nm) of flavonoid like substances in plant material (mistletoe tea bags), herbal mistletoe maceration at r.t. and within infusate. Rutin was used as standard.

Mistletoe from tea bags was analysed for its composition. Typical mistletoe proteins (VT, ML), triterpenoids (OA, BA, bAA, LA) and phenylpropane derivatives (SY, SYA) were quantified within the plant material (Table 1). The previously reported quantification methods used were partially optimized as described. Using 2.6 μm HPLC-material improved the separation of analytes as expected. The amount of VT quantified within the plant material (Fig. 1) was slightly lower than that published for mistletoe from apple trees which contained 0.2–0.8 g/100 g dry mass [14]. Furthermore, the authors quantified the amount of ML isoforms ML I, ML II and ML III and found 0.1–0.6 g/100 g within the dried plant material [14], whereas ML I was quantified in our study at 0.06 g/100 g. The OA and BA content of the plant material accords with previously published values for other dried mistletoe plant materials [13, 15], but the measured amount of bAA and LA was higher than found previously (<0.06 g/100 g within dried mistletoe from apple trees) (Fig. 2) [16]. The known amounts of SY and SYA range between 0.02 and 0.31 g/100 g respectively [17]. Thus, the amounts we found seem to be of the right order (Fig. 3). Flavonoids have been isolated from mistletoe before [18, 19], but no methods for their quantification have been published. Therefore, flavonoids were quantified using the Ph. Eur. Method for flavonoids in St. John’s wort dry extract, and all peaks within the mistletoe chromatogram at 360 nm were calculated as rutin (Fig. 4, Tabble 1).

Herbal mistletoe tea can be prepared as infusate or as a 12 h macerate at r.t. [2, 3]. Therefore, both preparation techniques were compared resulting in visually similar greenish brown extracts. A medium-sized cup was measured to contain 200 g, so the extraction of one tea bag results in a drug to solvent ratio of 1:10 as recommended by Madaus [2]. The analytes described above were quantified within the extracts, and the extraction power was calculated as the percentage of extracted compounds in relation to the amount within the dried drug (Table 2). Three independent (six for the triterpenoid determination within hot infusion) extracts were prepared respectively and the amounts of analytes are expressed as average over these different extracts. Standard deviations are thus higher than expected from the precision of the analysis methods.

VT were extracted neither by infusion nor by maceration. Because of their basic PI, their solubility rises with acid pH values of the solvent [20]. A phosphate buffered mistletoe extract (drug to solvent ratio 1:50; pH 7.5) contained 40 μg/mL VT A2 [21], and the total amount of VT (A1, A2 and A3) in a 1:10 phosphate buffered (pH 7.5) mistletoe extract was reported to be 244 μg/mL [22]. Therefore, tap water seems to be an insufficient extraction medium for VT.

ML is extracted by maceration at r.t. with an extraction efficiency of 43.0%, whereas it is not detectable within infusates (< 0.01 μg/mL). By briefly heating (boiling) the macerate it was shown that ML disappeared. Even though the maximum temperature within the infusate was below 100°C it is very likely that heat degradation is responsible for the lack of ML within the infusate as reported by Park et al. [23]. The relevance of an oral administration of 0.51 mg ML in one cup of macerate is questionable. By feeding up to 125–500 mg/kg body weight ML daily to mice, several identifiable changes in the morphology of non-Hodgkin lymphoma including the reduction of tumour mass were observed, thus demonstrating the oral availability of ML at a much higher dose than possible by herbal tea consumption [24]. However, the daily consumption of ML enriched herbal mistletoe tea by eight healthy volunteers generated ML antibodies as an immune response (ML dose not given) [24].

OA and BA are contained in low amounts within both herbal teas (Fig. 2) but the infusate contained more triterpene acids than the macerate (Table 2). Because < 1% of the triterpene acids are dissolved by maceration, a higher amount within the infusate could be explained by its temperature dependent solubility. Even so, the measured amounts accord with published data for triterpene acids in aqueous mistletoe extracts [13], and the total amount of OA and BA in one cup of mistletoe infusate is 0.174 mg. Oral absorption of OA (Cmax 66–74 ng/mL plasma) and BA (1.2 μg/mL plasma) occur probably by passive diffusion, and at much higher dosage (OA 50 mg/kg; BA 2.6 mg/kg) [25–27]. Thus the relevance of triterpene acids in herbal teas should be investigated in detail. Triterpene acetates are extracted with a higher extraction efficiency than triterpene acids, but no difference could be found between the infusate and the macerate (Table 2). Buffered mistletoe extracts have shown enhanced solubility of triterpene acetates in comparison to triterpene acids [16]. The acetates have only one polar molecule site, namely at C3, so possibly micelles may be formed as has been observed for cholesterol (critical micelle concentration 0.011–0.018 μg/mL) [28, 29].

SY and SYA were completely dissolved in both the infusate and the macerate, demonstrating the solubility and heat stability of these substances (Fig. 3). The calculated extraction power of 93.3–105.3% is in the analytical range of a complete extraction (standard deviation: 2.7–7.3%).

Flavonoid standards and a quantification method for mistletoe flavonoids were not available, so flavonoids and similar substances were quantified as rutin within a fingerprint chromatogram at 360 nm (Ph. Eur. monograph St. John’s wort dry extract, quantified). Using a four-wavelength detector, it was not even possible to determine peak spectra for flavonoid identification. Flavonoid-like substances were extracted by infusion at 43.3%, whereas less was dissolved by maceration at r.t. (31.4%). However, the fingerprint profile of macerate has generally smaller peaks than the infusate, but the peak at 16 min shows the reverse.

The extraction profile of constituents differs depending on the preparation of herbal mistletoe tea as infusate or macerate at r.t. Hot infusion degrades ML whereas the solubility of flavonoids, OA and BA rise with the extraction temperature. Both ML and triterpene acids can be absorbed orally, so the physiological relevance of low amounts from these substances within herbal tea preparations has to be investigated in detail. This study determines some differences between both extraction methods on the profile of solved substances. Because actives for the treatment of cardiovascular diseases with traditional herbal mistletoe tea are unknown and may well be left out during this study, no extraction method could be determined as superior. Anyhow these results may be of value for further research on the efficacy of herbal mistletoe tea.

## Experimental

Infusion of herbal mistletoe tea was compared with maceration at r.t. with respect to its content of mistletoe marker substances. Therefore, the amount of ML, VT, OA, BA, bAA, LA, SY, SYA and flavonoid like substances (flavonoid fingerprint at 360 nm) were quantified in the plant material and within the different herbal tea preparations in order to characterize the completeness of extraction and quantify the differences between these preparation methods. Validated and published methods were used or adapted as described.

### Plant material

Dried mistletoe herb within tea bags 1.94 g ± 0.06 g (± standard deviation, n = 3) (batch: 0906388) were a donation of Kneipp-Werke, Würzburg, Germany; a voucher specimen is archived there. The quality complies with Monograph of DAB 1996.

### Infusion

Mistletoe tea bags were infused with 200 g boiling tap water (23.3°dGH, total hardness of water according to German standards, Niefern-Öschelbronn, Germany) respectively and extracted for 5 min while moving the tee bags up and down. This was done in triplicate.

### Maceration

Six mistletoe tea bags were extracted each with 200 g cold tap water and for 12 h at r.t. while moving the tea bags up and down at the beginning and at the end of the period for a duration of 5 min in each case. After the tea bags were removed, three macerates were brought to boil for 1 min to study heat degradation effects.

### Quantification of analytes within plant material and extracts

VT isoforms A1, A2 and A3 were quantified by HPLC according to Winkler et al. by direct injection of filtered (Millex–HV 0.45 μm, Millipore, Schwalbach, Germany) herbal tea preparations [22]. A sample clean up was applied for the determination of VT within the plant material. Therefore 0.5–2.5 g of plant material per 20 mL acetic acid (2%, v/v) were macerated overnight [14], filtered (Millex–HV 0.,45 μm, Millipore, Schwalbach, Germany) and applied to solid phase extraction (1 mL extract per 500 mg Chromabond PCA, Machery-Nagel, Düren, Germany). The sample was eluted with acetic acid/methanol/water (12/5/50 v/v/v) [20,30]. The HPLC method was modified for an improved separation on 2.6 μm spherical material (Kinetex 2.6 μm C18 100 Å, 4.6 x 100 mm, Phenomenex, Aschaffenburg, Germany): 100 μL of standard or sample solution were injected and separated by a gradient elution from 5% acetonitrile in water to 38% acetonitrile (containing 0.1% trifluoracetic acid respectively) at 1.5 mL/min within 40 min. UV detection at 210 nm and external standard calibration with VT A2 (purity > 90% [21]) was applied for all measured VT isoforms (limit of quantification (LOQ): 1μg/mL within the measuring solution) [21, 22].

ML were quantified by enzyme-linked immunosorbent assay (ELISA) following the method published by Manjolovic et al. [31]. For quantitative determination of ML in plant material, 0.5–2.5 g of plant material per 200 mL sodium phosphate buffer (138 mM, pH 7.5) were macerated overnight [14]. This extract and the herbal tea preparations were filtered (Millex–GV 0.22 μm, Millipore, Schwalbach, Germany) and diluted for ELISA measurement.

OA, BA, bAA and LA were quantified in triplicate within the plant material and the different herbal tea preparations by GC-FID [13]. BA and OA (each >97%, Extrasynthese, Genay Cedex, France) were used as external standards and the triterpene acetates were quantified as OA. According to the method (LOQ 21 μg/mL within the measuring solution) 0.07 g/100g was the LOQ for the plant material and 0.21 μg/mL for the herbal tea preparation. The recovery rate of internal standard cholesterol (Carl Roth, Karlsruhe, Germany) was 99% ± 4% (n = 18).

SY and SYA were quantified following the sample preparation and clean up procedure published by Krüger and Frank [17]. Their HPLC separation was improved on the 2.6 μm spherical material Kinetex described above: 5 μL of SY standard at 0.443 mg/mL in methanol/water v/v (>99%, Carl Roth, Karlsruhe, Germany) or sample solution were injected on the column and separated by a gradient from 5% acetonitrile in water to 16% acetonitrile (containing 0.1% trifluoracetic acid respectively) at 1.5 mL/min within 25 min. UV detection at 268 nm and external standard calibration with SY was applied for SY and SYA (LOQ: 1 μg/mL within the measuring solution). The recovery rate of internal standard phenacetin (98%, Fluka, Taufkirchen, Germany) was 96% ± 4% (n = 14).

Mistletoe flavonoid standards and a quantification method for mistletoe were not available, so flavonoid like substances were quantified within a fingerprint chromatogram at 360 nm. Therefore the European monograph for St. John’s wort dry extract, quantified (Ph. Eur. 6) was used. Briefly, extracts were separated using the HPLC Kinetex column above and all peaks between 2.5 and 19 min (360 nm) were quantified as rutin (USP reference standard, Basel, Switzerland).

### Statistics

Microsoft Excel was used for the calculation of the mean and standard deviation. Values are expressed as mean ± standard variation with the number (n) of independent experiments given.

## Figures and Tables

**Tab. 1.** Quantitative characterization of plant material (mistletoe tea bags).

**Analyte**	**Amount [g/100 g]**
viscotoxins (VT) sum of VT A1, A2, A3 quantified as VT A2	0.11 ± 0.01 (n = 5)
mistletoe lectin (ML)	0.06 ± 0.01 (n = 5)
oleanolic acid (OA)	0.751 ± 0.021 (n = 3)
betulinic acid (BA)	0.088 ± 0.003 (n = 3)
β-amyrin acetate (bAA) quantified as OA	0.167 ± 0.003 (n = 3)
lupeol acetate (LA) quantified as OA	0.137 ± 0.003 (n = 3)
syringin (SY)	0.036 ± 0.002 (n = 3)
syringenin apiosylglucoside (SYA) quantified as SY	0.038 ± 0.002 (n = 3)
flavonoid like substances quantified as rutin	0.113 ± 0.011 (n = 3)

±…standard deviation of n independent analysis; n…is the number of independent analysis.

**Tab. 2.** Quantitative characterization of herbal mistletoe teas as infusate and maceration at r.t. (2 g / 200 g).

**analyte**	**infusate**		**maceration at r.t.**	

	**amount [μg/mL]**	extraction efficiency* [%]	**amount [μg/mL]**	extraction efficiency* [%]
viscotoxins (VT) sum of VT A1, A2, A3 quantified as VT A2	< LOD (n = 3)	n.a.	< LOD (n = 3)	n.a.
mistletoe lectin (ML)	< 0.01	n.a.	2.57 ± 0.09 (n = 3)	43.0
oleanolic acid (OA)	0.71 ± 0.10 (n = 6)	0.9	0.47 ± 0.10 (n = 3)	0.6
betulinic acid (BA)	0.16 ± 0.04 (n = 6)	1.8	0.08 ± 0.02 (n = 3)	0.9
β-amyrin acetate (bAA) quantified as OA	1.37 ± 0.16 (n = 6)	8.2	1.27 ± 0.22 (n = 3)	7.6
lupeol acetate (LA) quantified as OA	0.69 ± 0.10 (n = 6)	5.1	0.61 ± 0.10 (n = 3)	4.4
syringin (SY)	3.51 ± 0.34 (n = 3)	94.8	3.45 ± 0.21 (n = 3)	93.3
syringenin apiosylglucoside (SYA) quantified as SY	4.01 ± 0.34 (n = 3)	105.1	4.01 ± 0.30 (n = 3)	105.3
Flavonoid like substances quantified as rutin	4.91 ± 0.25 (n = 3)	43.3	3.56 ± 0.12 (n = 3)	31.4

* …Extraction efficiency is the percentage of extracted compound in relation to the amount in the dried drug; ±…standard deviation of n independent analyses; n…is the number of herbal teas being prepared for quantification of analytes; n.a. …not applicable.
